# Supplementary material for: Predicting visual function by interpreting a neuronal wiring diagram
Source: Nature. 2024 Oct 2;634(8032):113–23. doi: 10.1038/s41586-024-07953-5 (PMC11446822; doi:10.1038/s41586-024-07953-5)
Supplement: Supplementary file 6 — CRF and ERF predictions for individual TmY4 and TmY9 cells. Analogous to Supplementary Data 3, but for TmY target types. Shown are the top four monosynaptic pathways, the strongest pathway passing through each of the top ten intermediary types (ranking from Extended Data Fig. 7), and the trisynaptic pathway Tm1–TmY–Dm3–TmY (see the section entitled Prediction of spatial normalization). [file 41586_2024_7953_MOESM6_ESM.zip › DataS4/TmY9q/720575940631234105.pdf]

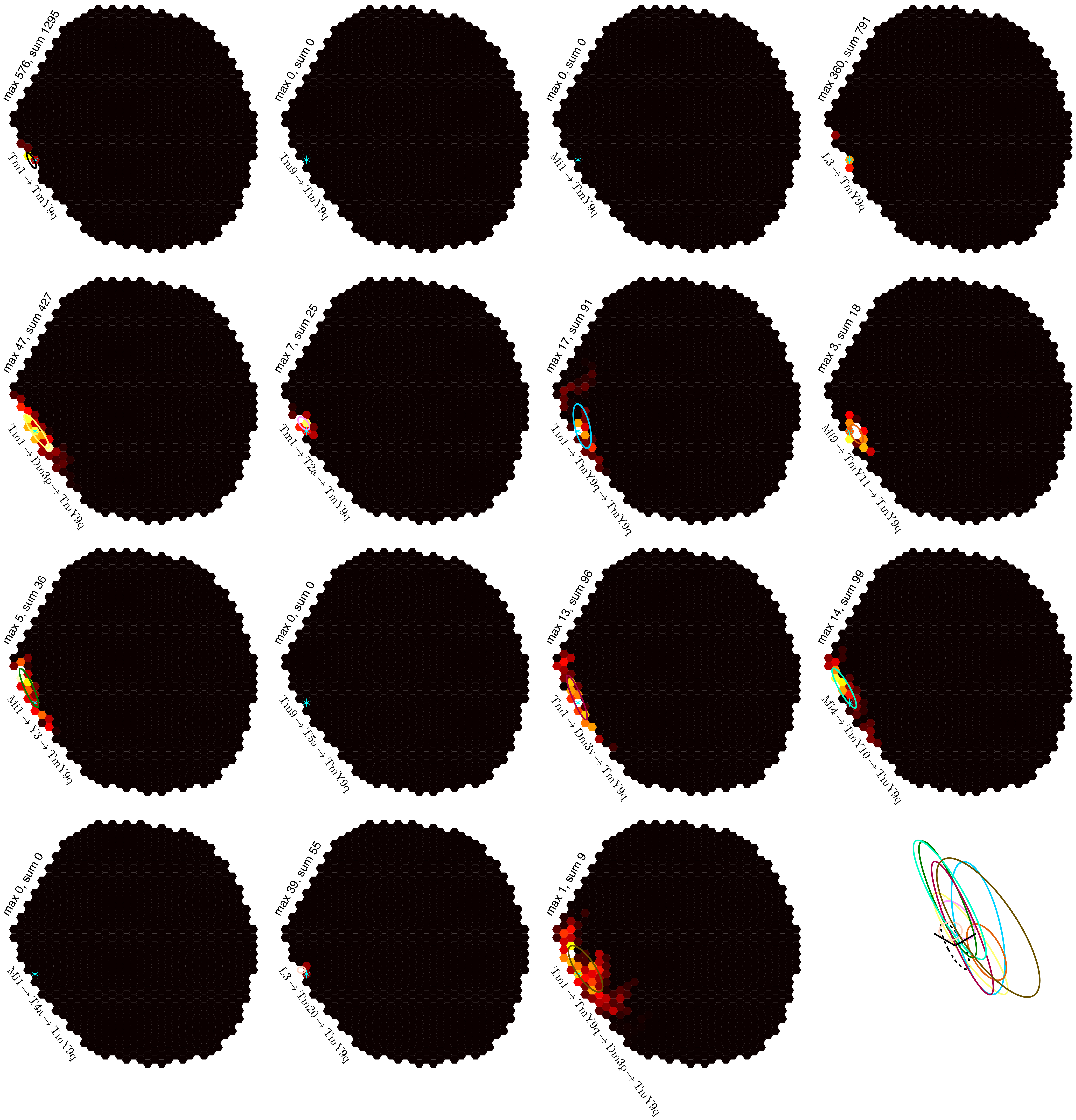

max 576, sum 1295
Tm1 → TmY9q
max 0, sum 0
Tm9 → TmY9q
max 0, sum 0
Mi1 → TmY9q
max 360, sum 791
L3 → TmY9q
max 47, sum 427
Tm1 → Dm3p → TmY9q
max 7, sum 25
Tm1 → T2a → TmY9q
max 17, sum 91
Tm1 → TmY9q → TmY9q
max 3, sum 18
Mi9 → TmY11 → TmY9q
max 5, sum 36
Mi1 → Y3 → TmY9q
max 0, sum 0
Tm9 → T5a → TmY9q
max 13, sum 96
Tm1 → Dm3v → TmY9q
max 14, sum 99
Mi4 → TmY10 → TmY9q
max 0, sum 0
Mi1 → T4a → TmY9q
max 39, sum 55
L3 → Tm20 → TmY9q
max 1, sum 9
Tm1 → TmY9q → Dm3p → TmY9q
